# Chrysin's protective effect on the expression of *protamine*, *Tsga10*, *Dazl*, and *Akap4* genes against diazinon toxin in male rats: An experimental study

**DOI:** 10.18502/ijrm.v23i3.18778

**Published:** 2025-06-10

**Authors:** Fatemeh Mojibi, Esmail Fattahi, Seyed Gholam Ali Jorsaraei, Sohrab Kazemi, Maryam Gholamitabar Tabari

**Affiliations:** ^1^Department of Biology, Ayatollah Amoli Branch, Islamic Azad University, Amol, Iran.; ^2^Faculty of Medicine, Babol University of Medical Sciences, Babol, Iran.; ^3^Cellular and Molecular Biology Research Center, Health Research Institute, Babol University of Medical Sciences, Babol, Iran.; ^4^Health Reproductive Research Center, Sari Branch, Islamic Azad University, Sari, Iran.

**Keywords:** Diazinon, Chrysin, Spermatogenesis, Rat male, Gene expression.

## Abstract

**Background:**

Environmental pollution due to pesticides is a major health problem worldwide that causes toxicity. Chrysin (C) is a flavonoid that reduces the adverse effects caused by diazinon (D).

**Objective:**

To investigate the protective effects of C on the expression of *protamine*, testis-specific gene antigen 10 (*Tsga10*), deleted azoospermia-like gene (*Dazl*)*,* and A-Kinase anchoring protein 4 (*Akap4*) genes in the testicular tissue of rats receiving D.

**Materials and Methods:**

In this experimental study, 42 Wistar rats (7–8 wk, 180–250 gr) were selected. This study was conducted in 2021 at the Pasteur Institute of Iran, Amol Research Center, Amol, Iran. Injections were performed 5 days a week for 4 wk. Random grouping of rats: 1) control: without any injection. 2) Sham: injection of 10% tween 80 solution as D solvent. 3) D: injection at a concentration of 20 mg/kg/BW. 4 and 5) C with low dose (C10 mg/kg/BW) and high dose (C20 mg/kg/BW). 6 and 7) D with low dose of C (D20 mg/kg/BW/ C10 mg/kg/BW) and D with high dose: (D20 mg/kg/BW/ C20 mg/kg/BW). Finally, the testes were examined in terms of histology and gene expression.

**Results:**

Histological results showed that D improved spermatogenesis after C intervention by reducing germinal cells, diameter, and lumen area. SPSS results showed that D reduced the expression of *Dazl *(p < 0.0001), *Tsga10* (p = 0.01)*,* and *protamine *(p < 0.0001) genes, but increased the expression of *Akap4* gene (p < 0.0001).

**Conclusion:**

D has a negative effect on the expression of meiotic genes in rat testicular tissue, while C greatly reduces the adverse effects caused by D.

## 1. Introduction 

Environmental pollution by pesticides, especially organophosphate insecticides, which account for 70% of insecticides used, is a global health problem. Diazinon (D), also known as an organophosphate insecticide, is used in agriculture to control pests and protect crops (1). Recent studies have shown that the reproductive system is the main target of D toxicity, but the cellular and molecular mechanisms by which D affects sperm are still poorly understood. Therefore, there is a need for further research to investigate the underlying mechanisms by which D causes male fertility disorders and infertility (2).

In recent years, global attention has increased to the importance of medicinal plants in traditional medicine. Extracts obtained from these plants and other natural sources contain a variety of molecules with strong biological activities. Flavonoids are a group of natural compounds widely found in plants and comprise a large group of low molecular weight polyphenolic compounds. Chrysin (C, 5,7-dihydroxyflavone) is a natural flavone found in honey, propolis, and various medicinal plants (3) which includes a range of biological functions, including anticancer, antimicrobial, anti-inflammatory, antiallergic, antioxidant, antiviral, hepatoprotective and neuroprotective functions (4). The beneficial effects of C are thought to be due to its ability to scavenge free radicals (5). As a result, this extract may affect and reduce fertility.

Previous studies have reported the antioxidant effects of C on spermatogonia stem cells isolated from mouse neonates. It was found that C at low concentrations can have a protective effect on mouse spermatogenic stem cells and exerts its effect by increasing viability, reducing cell apoptosis, and inhibiting free radicals (6, 7). Given the widespread use of pesticides in agriculture and the known effects of organophosphates on the human reproductive system as well as the lack of evidence of the precise effects of C on semen quality and spermatogenesis in animals poisoned with organophosphates, this study investigated the protective effects of C at different concentrations on the process of spermatogenesis in rats receiving D. In addition, the aim and novelty of our study in this project was to answer the main question of whether excessive use of this pesticide for citrus and rice fields, which is sometimes used in a non-standard way, can affect gene expression in the long term?

There are specific genes in the testes, called chauvinist genes, that play an important role in the process of spermatogenesis (8). The autosomal gene deleted in the deleted azoospermia gene (*DAZL*) is a member of the chauvinist genes that play an important role in reproduction and differentiation of germ cells. The testis-specific antigen 10 gene (*Tsga10*) is one of the testis-specific genes that is expressed in the last stages of spermatogenesis and plays an important role in the fibrous coating of the sperm tail (9). Moreover, *protamines* are unique proteins of sperm that package and protect the hereditary chromatin until fertilization (10). In addition, A-Kinase anchoring protein 4 (*Akap4*) is expressed in the post-meiotic stage of spermatogenesis and produces many proteins in the fibrous sheath of sperm flagella (11).

Hence, this study focused on genes involved in spermatogenesis, the expression of which is related to spermatogenesis and infertility and has not been studied much. Considering the role of polyphenolic compounds as therapeutic agents, the present study investigated the protective effect of C at different concentrations on the expression of *protamine*, *Tsga10*, *Dazl,* and *Akap4* genes against D toxin in male rats.

## 2. Materials and Methods

### Chemical materials

Diazinon (CAS-Nummer: 480–40-0), Chrysin (CAS-Nummer: 333–41-5), and tween 80 (CAS-Nummer: 9005–65-6) were purchased as solvents for CHR from Merck, Germany. Also, an RNA extraction kit was obtained from Macherey-Nagel (MN), a cDNA synthesis kit (Cat-Number: 4387406) from Applied Biosystems Inc (ABI), and Master Mix SYBR Green kit (Cot.NO: A325402) was prepared from Ampliqon (Denmark).

### Animal issues

In this experimental study, 42 male Wistar rats (7–8 wk, 180–250 gr), were purchased from Pasteur Institute of Iran, Amol Research Center, Amol, Iran and transferred to the animal room. Rats were housed at 22 C ( ± 2 C) and 55% relative humidity, in a 12-hr light/dark cycle with free access to food and water for 1 wk for acclimatization. This study was conducted at the Pasteur Institute of Iran, Amol Research Center, Amol, Iran in 1400.

### Induction of D

After adaptation, the rats were randomly divided into 7 groups (n = 6/each). Injections were performed 5 days a week as intraperitoneal injection (IP) for 4 wk and the rats rested for 2 days. The grouping and injection of D was as follows: 1) control group (Cnt): the group in which injection was not performed. 2) Sham group (Sham): received 10% tween 80 solution as D solvent. 3) D experimental group: the group that received D at a concentration of 20 mg/kg/ body weight (BW) (1).

### C injection

C was administered after the completion of D injection in the treatment groups (groups 4, 5, 6 and 7) as follows: C injection in the treatment groups (4 and 5) is as follows: 4) experimental group C10: (C at a dose of 10 mg/kg/BW) and 5) experimental group C20: (C at a dose of 20 mg/kg/BW). After that, D was injected at a dose of 20 mg/kg/BW and high and low doses of C in treatment groups (6 and 7) as follows: 6) experimental group (D20C10): the group that received C at a concentration of 10 mg/kg/BW and D at a concentration of 20 mg/kg/BW. 7) experimental group (D20C20): C at a concentration of 20 mg/kg/BW and D at a concentration of 20 mg/kg/BW (12).

### Histological analysis

1 wk after the last injection, the rats were euthanized after anesthesia with ether. Then, in the shortest time, the skin of the scrotum was waxed and sterilized, and the testicles were removed. The right testicle was considered for histological examination. Testicular tissue samples were placed in Boen's solution at room temperature for 24 hr to fix completely.

Also, the epididymis was separated from the testes and placed in 5 ml of T6 culture medium (Tofiq Daro, Iran) containing bovine serum albumin for 1 hr at 37 C in a CO_2_ incubator. For this purpose, tissue samples were fixed in 10% formalin. Then, it was cut with a thickness of 5 µm and stained with a hematoxylin and eosin staining solution (13). After washing with phosphate buffer saline PBS, the left testicle was placed in cryovials and kept at 80 C until the experiments.

### RNA extraction, cDNA synthesis, and real-time polymerase chain reaction (RT-qPCR) assay

To examine the expression of the studied genes, the RT-qPCR technique was used. First, primers were designed and total RNA was extracted from the tissues and converted to cDNA (Table I). Then, cDNA was amplified by PCR and quantitative expression of the genes was confirmed by RT-qPCR. Determining the relative value in real-time PCR was done by measuring the increase in fluorescence light as a result of the binding of SYBR green dye. During this step, a polymerase chain reaction was performed for cDNA samples for *protamine*, *Tsga10*, *Dazl*, and *Akap4* genes of testicular tissue using a Cybergreen kit (Ampliqon, Denmark) on a Corbett 6000 Gene Rotor machine. The expression ratio of the examined genes was evaluated using the threshold cycle (CT) comparative method. After examining the CT values obtained from the biological and technical replicates of each treatment, the average CT for the technical replicates of *protamine*, *Tsga10*, *Dazl,* and *Akap4* genes of the testis tissue was calculated, followed by the data. Each specific gene was calculated by its expression ratio. The expression level of the desired genes was calculated by the Livak method (2- ΔΔ CT). A specific standard curve for each gene (at least 5 logarithmic concentrations) was drawn relative to the positive control. The expression level of the target gene was considered normalized with the reference gene. In addition, the expression of the Cnt group genes was considered as calibrators. Finally, the specificity of the reactions was evaluated by analyzing the melting and diffusion curves.

### Body and testicle weight measurement

The weight of the body and testicles of the rats in each group were measured in the first week and 4 ${}^{\text{th}}$ wk after the treatment using a digital scale.

**Table 1:** List of primers used for RT-PCR process

**Genes**	**Access number**	**Sequence (5 '→ 3 ' )**	**Product length (bp)**
*Protamine*	NM_012873.1	F-5 ' - AGAGCGCGTGGAGGACTATG -3 '	158
		R-5 ' - TTCTGCAGCCTCTGCGATG -3 '	
*Tsga10*	NM_001393709.1	F-5 ' - GGCTCACTTGGAACAGCGGATAG -3 '	161
		R-5 ' - TCTCGGTGTCCATTGCCTTTCTTG -3 '	
*Dazl*	NM_001109414.1	F-5 ' - CATCAGCAACCACCAGTCAAG -3 '	113
		R-5 ' - GAAACTCCTGATTTCGGTTTCATC -3 '	
*Akap4*	NM_024402.1	F-5 ' - GACAACAAGATCAGGACCGAAAAG -3 '	195
		R-5 ' - GGCATACAGATCCCTCCGTC -3 '	
RT-PCR: Real-time polymerase chain reaction, *Tsga10*: Testis-specific gene antigen 10, *Dazl*: Deleted azoospermia-like gene, *Akap4*, A-Kinase anchoring protein 4, bp: Base pair, F: Forward, R: Reverse			

### Ethical Considerations

All steps in this project were approved by the Animal Care and Ethics Committee of the Islamic Azad University of Babol branch, Babol, Iran (Code: IR.IAU.BABOL.REC.1399.032). Ethical instruction guidelines for working with laboratory animals were carried out in accordance with the ARRIVE guidelines.

### Statistical Analysis

To analyze the data, in the descriptive statistics section, the standard deviation, mean, and graph dispersion indices were used. In the inferential statistics section, the normality of the data distribution was examined using the Kolmogorov-Smirnov test and the equality of variances was examined using the Levine test. To determine the significance, a one-way analysis of variance test was used, and in case of significance, to determine the significant difference between the groups, the post hoc LSD test was used at the significance level of p ≤ 0.05. GraphPad Prism version (8.4.3) software was used for data analysis, and all statistical operations were performed using IBM SPSS Statistics 23 software.

## 3. Results

### C improves the diameter of the lumen and the epithelium of the spermatogenic tubes

**Figure 1:**
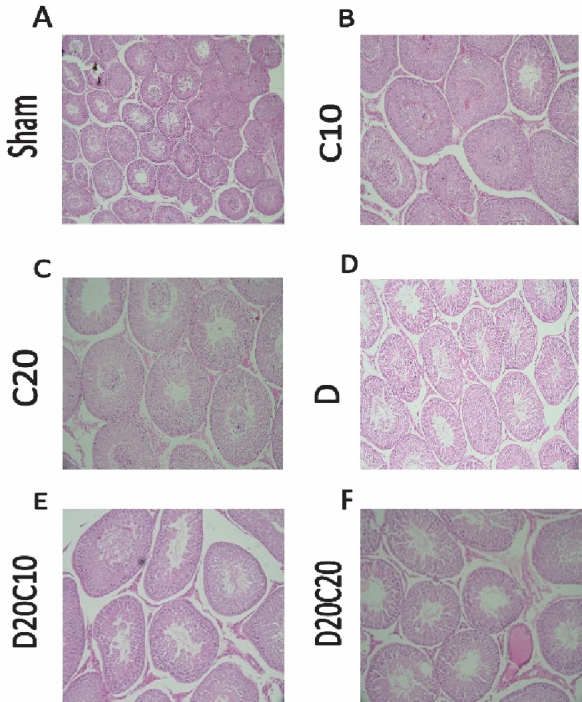
Photomicrograph of rat testis stained with hematoxylin-eosin. A) Sham group, B) C10 (Chrysin 10 mg/kg), Chrysin, C) C20 (Chrysin 20 mg/kg), D) D (Diazinon), E) D20C10 (Diazinon 20 mg/kg and Chrysin 10 mg/kg), F) D20C20 (Diazinon 20 mg/kg and Chrysin 10 mg/kg) ( × 400 magnification).

The results of the photo microgram of the testes of rats stained with hematoxylin and eosin (Figure 1) show that in C10 (Figure 1B) and C20 (Figure 1C) groups, the diameter and area of the lumen and the diameter of the epithelium of the spermatogenic tubules are completely normal. Still, in the D group (Figure 1D), due to the destruction of germ cells, the diameter and area of the lumen increased compared to other groups, and the diameter of the epithelium decreased compared to other groups. In addition, C was able to somewhat reduce the diameter and area of the lumen in the D20C10 (Figure 1E) and D20C20 (Figure 1F) groups compared to the D group and increase the diameter of the epithelium compared to the D group.

### BW and testicle changes

Evaluation of the average initial weight and the last day's BW of the rats showed that no significant difference was observed between the 7 groups. Examining the average weight of the testis in the D20C20 group compared to the Cnt (p = 0.03), Sham (p = 0.01), and D (p = 0.03) groups had decreased, which was statistically significant. In addition, the examination of the difference in the average ratio of testis weight to BW showed that a significant difference was observed between the Sham (p = 0.04) and Cnt (p = 0.05) groups and the D20C20 experimental group. In contrast, no significant difference was observed between the other groups (Table II).

### C increased tubular differentiation (TDI), repopulation (RI), and spermiogenesis (SPI) 

The results of the ANOVA test related to SPSS software showed that the average spermatogenesis in the D group compared to the sham and Cnt groups (p < 0.001) decreased significantly. In the experimental groups, D20C20 (p < 0.001) and D20C10 (p = 0.023) increased significantly compared to the D group, while no significant difference was observed in other groups. In addition, examination of the average tubular differentiation showed that in the D group, the average TDI decreased significantly compared to the Sham and Cnt groups (p < 0.001), if the TDI in the intervention groups D20C20 (p < 0.001) and D20C10 (p = 0.002) increased compared to D. Also, the results of the average regeneration coefficient showed that in the D group, the RI was significantly reduced compared to the Sham and Cnt groups (p < 0.001). This reduction was significantly moderated by using the D20C20 (p = 0.003) and D20C10 (p = 0.050) treatment groups compared to the D group, no significant difference was observed in other groups (Table III).

### C restored the testicular epithelium

The results of the testicular epithelium were measured using SPSS statistical analysis in the Cnt and intervention groups. The results show that no statistically significant difference was observed in the tubular diameter and tubular area groups, which can be concluded that the interventions do not affect the diameter and area of the sperm tubes. The results of epithelial height show that it has decreased significantly in the D (p = 0.004), D20C20 (p = 0.05), and D20C10 (p = 0.04) groups compared to the Cnt group. The luminal diameter results show that a significant increase was observed in the D group (p = 0.03) compared to the Cnt group. In contrast, the luminal diameter in the D20C20 (p = 0.02) and D20C10 (p = 0.03) groups was reduced compared to the D group, which can indicate the effect of interventions in the improvement process. In addition, the luminal area showed a significant increase in the D group (p = 0.002) compared to the Sham and Cnt groups. In contrast, in the intervention groups, this increase was moderated, but the difference was not statistically significant (Table IV).

### Modulation of *Dazl* gene expression after C intervention

**Figure 2:**
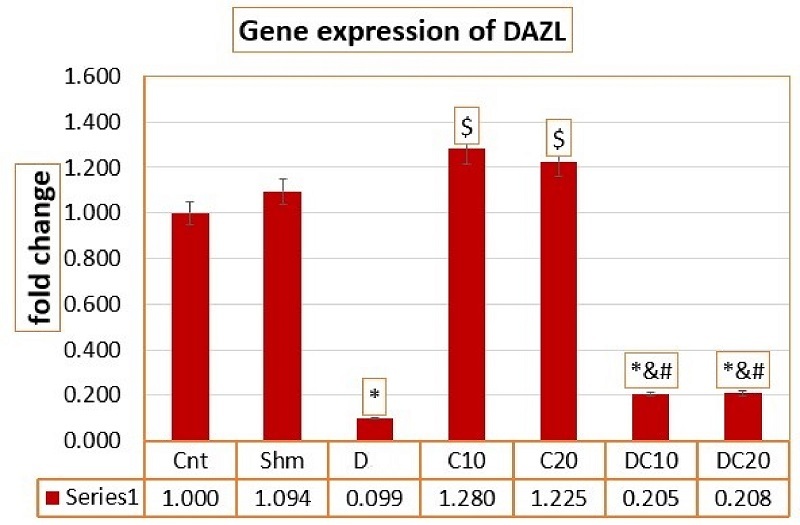
Average expression of the *Dazl* gene in groups. *Significant difference compared to the control group, ${}^{\$}$ Significant difference compared to the D 20 group, ${}^{\&}$ Significant difference compared to the C 10 group, ${}^{\#}$ Significant difference compared to the C 20 group. Cnt (Control group), Shm (Sham group), Diazinon (D) (Diazinon group), Chrysin (C) C10 (Chrysin with 10 mg/kg), C20 (Chrysin with 20 mg/kg), D20C10 (Diazinon 20 mg/kg and Chrysin 10 mg/kg), D20C20 (Diazinon 20 mg/kg and Chrysin 20 mg/kg).

The results of Prism graph analysis to find out the mean of the *Dazl* gene show that this gene has decreased compared to the Cnt and Sham groups (p < 0.0001). Groups treated with C and D moderated the decrease in gene expression in such a way that gene expression in the C10 and C20 groups increased compared to the Cnt group and increased compared to the D group, which was statistically significant (p < 0.0001). Also, gene expression in the intervention groups with D20C10 and D20C20 decreased compared to the Cnt group but increased compared to the D group, which were statistically significant differences (p < 0.0001). Which, in general, indicated the modulation of *Dazl* gene expression by using treatment groups (Figure 2).

### *Tsga10* gene expression changes after C intervention

**Figure 3:**
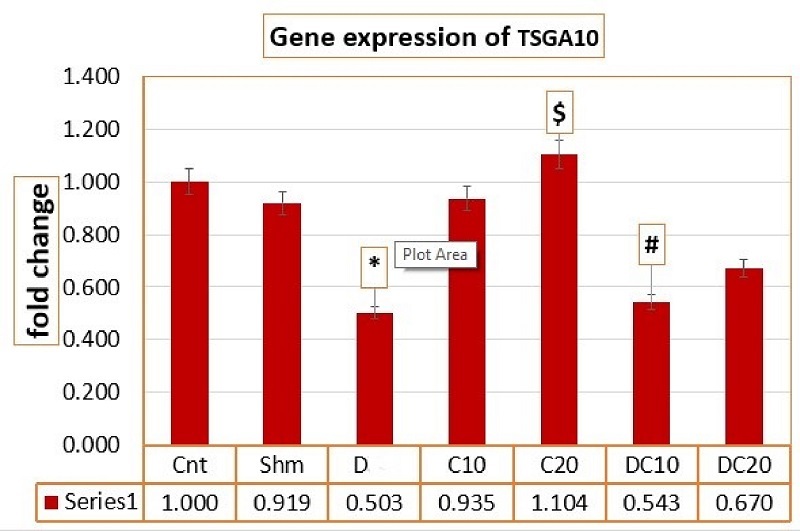
Average *Tsga10* gene expression in different groups. GraphPad Prism software version (8.4.3) was used for statistical analysis. *Significant difference compared to the control group, ${}^{\$}$ Significant difference compared to the D 20 group, ${}^{\&}$ Significant difference compared to the C 10 group, ${}^{\#}$ Significant difference compared to the C 20 group (p < 0.05). Cnt (Control group), Shm (Sham group), Diazinon (D) (Diazinon group), Chrysin (C) C10 (Chrysin with 10 mg/kg), C20 (Chrysin with 20 mg/kg), D20C10 (Diazinon 20 mg/kg and Chrysin 10 mg/kg), D20C20 (Diazinon 20 mg/kg and Chrysin 20 mg/kg).

The results of the ANOVA test for the average expression of the meiosis gene *Tsga10* in different research groups, the calculated F value (4.410), and its significance at p = 0.002 indicate a significant difference in the average expression of the meiosis gene *Tsga10* between the groups. Different, the mean expression of the gene *Tsga10* in the C20 group was significantly decreased compared to the D group (p = 0.01). Also, the D20C10 groups had a significant decrease compared to the C20 group (p = 0.02) (Figure 3).

### C/D modulates the expression of *Akap4* gene

**Figure 4:**
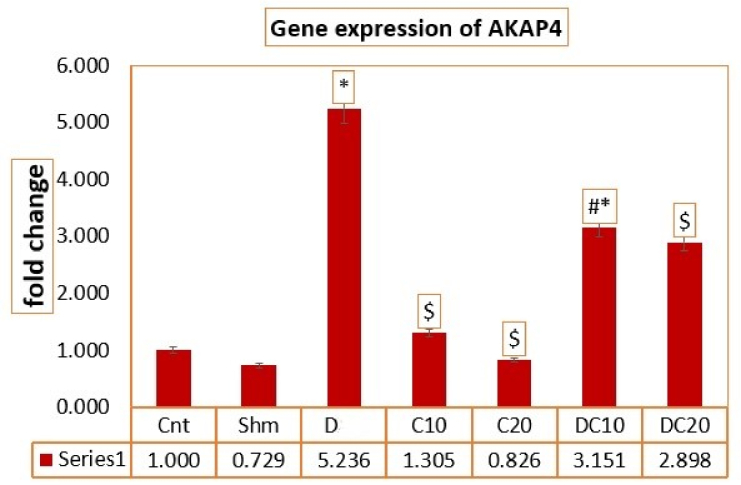
Average *Akap4* gene expression in different groups. GraphPad Prism software version (8.4.3) was used for statistical analysis. *Significant difference compared to the control group, ${}^{\$}$ Significant difference compared to the D 20 group, ${}^{\&}$ Significant difference compared to the C 10 group, ${}^{\#}$ Significant difference compared to the C 20 group (p < 0.05). Cnt (Control group), Shm (Sham group), Diazinon (D) (Diazinon group), Chrysin (C) C10 (Chrysin with 10 mg/kg), C20 (Chrysin with 20 mg/kg), D20C10 (Diazinon 20 mg/kg and Chrysin 10 mg/kg), D20C20 (Diazinon 20 mg/kg and Chrysin 20 mg/kg).

The average expression value of *Akap4* gene F (11.662) and its significance at p < 0.0001 indicates a significant difference in the average expression of the *Akap4* gene using the ANOVA test. Among the different groups, the average *Akap4* gene expression in the D group increased compared to the Cnt and Sham groups (p < 0.0001), which caused gene expression modulation in the treatment groups. In this way, in the C10 (p < 0.0001), C20 (p < 0.0001) and D20C20 (p = 0.03) groups compared to D group (p < 0.05), *Akap4* gene expression has shown a more significant decrease, which indicates the modulation of gene expression by using C (Figure 4).

### C increases the expression of the *protamine* gene

**Figure 5:**
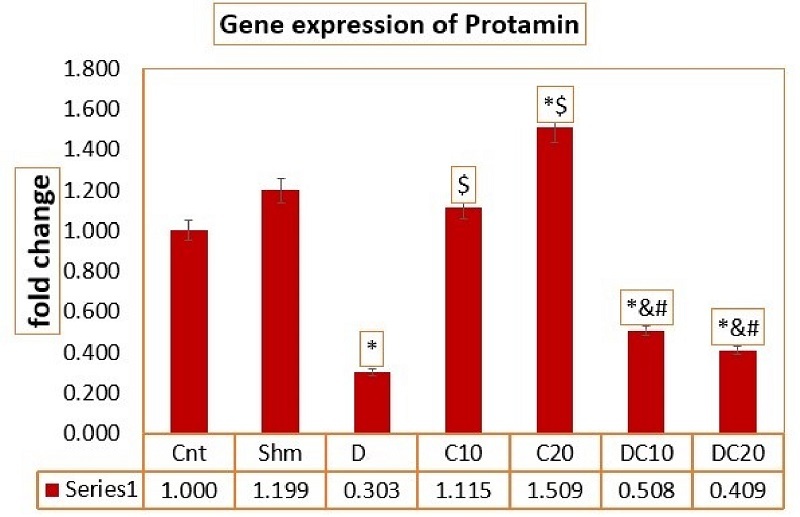
Average expression of the *Protamine* gene in different groups. GraphPad Prism software version (8.4.3) was used for statistical analysis. *Significant difference compared to the control group, ${}^{\$}$ Significant difference compared to the D 20 group, ${}^{\&}$ Significant difference compared to the C 10 group, ${}^{\#}$ Significant difference compared to the C 20 group (p < 0.05). Cnt (Control group), Shm (Sham group), Diazinon (D) (Diazinon group), Chrysin (C) C10 (Chrysin with 10 mg/kg), C20 (Chrysin with 20 mg/kg), D20C10 (Diazinon 20 mg/kg and Chrysin 10 mg/kg), D20C20 (Diazinon 20 mg/kg and Chrysin 20 mg/kg).

The results of the ANOVA test for the average expression of the protamine gene in different research groups, F value (20.132), show the average expression of the *protamine* gene among different groups. The average expression of the *protamine* gene in the D group was decreased compared to the Cnt and Sham groups (p < 0.0001). After treatment, *protamine* gene expression was increased. In the C10 and C20 groups, *protamine* gene expression showed a significant increase compared to the D20C10 and D20C20 groups (p < 0.0001). It can be concluded that the C treatment group was able to compensate for the decrease in *protamine* gene expression better than the D treatment groups (Figure 5).

**Table 2:** Effects of C and/or D on the relative body and testis weight in male Wistar rats

**Variables**	**First body weight (g)**	**Last body weight (g)**	**Testis weight (g)**	**Testes/body weight ratio (%)**
**Cnt**	204 ± 15.8	255 ± 17.5	1.63 ± 0.148	0.639 ± 0.041
**Shm**	203 ± 24.2	258 ± 21.6	1.66 ± 0.12	0.647 ± 0.082
**D**	204 ± 16.1	241 ± 17.7	1.51 ± 0.153	0.63 ± 0.08
**C10**	207 ± 21.8	265 ± 24.1	1.62 ± 0.112	0.616 ± 0.077
**C20**	205 ± 17.4	260 ± 16	1.64 ± 0.188	0.63 ± 0.07
**D20C10**	204 ± 19	246 ± 20.3	1.5 ± 0.118	0.616 ± 0.082
**D20C20**	206 ± 13.2	252 ± 17.5	1.45 ± 0.134*	0.577 ± 0.065*
**P-value**	1.00	0.35	0.05	0.04
Data were presented as Mean ± SD. The Kolmogorov-Smirnov test was used to determine the normality of the data distribution. To determine significance, one-way analysis of variance test and LSD post hoc test were used at a significance level of p ≤ 0.05. *P < 0.05. Cnt: Control group, Shm: Sham group, D: Diazinon (Diazinon group), C: Chrysin (C10, Chrysin 10 mg/kg), C20 (Chrysin 20 mg/kg), D20C10 (Diazinon 20 mg/kg and Chrysin 10 mg/kg), D20C20 (Diazinon 20 mg/kg and Chrysin 20 mg/kg)				

**Table 3:** Effects of C and/or D on TDI, RI, and SPI indices in male Wistar rats

**Variables**	**SPI (%)**	**TDI (%)**	**RI (%)**
**Cnt**	91.2 ± 2.04	95.3 ± 2.34	78.3 ± 3.67
**Shm**	91.8 ± 1.17	93.2 ± 1.94	79.2 ± 2.79
**D**	66.3 ± 4.68***	59.2 ± 5.56***	63.0 ± 3.58***
**C10**	93 ± 2.9	90.5 ± 2.07**	77.3 ± 2.8
**C20**	88.8 ± 3.49	88.2 ± 2.4***	75.5 ± 3.62
**D20C10**	71.2 ± 4.45 † ***	66.0 ± 4.82 † ***	66.8 ± 4.12 †† ***
**D20C20**	75.0 ± 4.29 †† ***	72.5 ± 4.23 ††† ***	69.3 ± 3.01 †† ***
**P-value**	< 0.001	< 0.001	< 0.001
Data were presented as Mean ± SD. The Kolmogorov-Smirnov test was used to determine the normality of the data distribution. To determine significance, one-way analysis of variance test and LSD post hoc test were used at a significance level of p ≤ 0.05. Significantly different from Cnt, **P < 0.01, ***P < 0.001, † Significantly different from D at, †† Significantly different from D at p < 0.01. ††† Significantly different from D at ***P < 0.001. Not significantly different from Cnt and D, p > 0.05. SPI: Spermatogenesis, TDI: Tubular differentiation, RI: Regeneration coefficient, Cnt: Control group, Shm: Sham group, D: Diazinon (Diazinon group), C: Chrysin (C10, Chrysin 10 mg/kg), C20 (Chrysin 20 mg/kg), D20C10 (Diazinon 20 mg/kg and Chrysin 10 mg/kg), D20C20 (Diazinon 20 mg/kg and Chrysin 20 mg/kg)			

**Table 4:** Effects of C and/or D on tubular and luminal parameters in male Wistar rats

**Variables**	**Tubular diameter (µm)**	**Tubular area (µm^2^)**	**Epithelial height (µm)**	**Luminal diameter (µm)**	**Luminal area (µm^2^)**
**Cnt**	246.7 ± 8.36	53788 ± 4886	71.0 ± 6.03	125.2 ± 9.39	12740 ± 974
**Shm**	254.0 ± 9.72	55831 ± 6176	72.0 ± 6.45	128.7 ± 9.63	13376 ± 1351
**D**	233.5 ± 22.69	47869 ± 8449	56.5 ± 7.99**	140.8 ± 18.43*	16177 ± 1491***
**C10**	243.0 ± 24.90	50014 ± 5235	69.0 ± 9.27	124.3 ± 7.76	12436 ± 1076
**C20**	242.2 ± 24.17	48961 ± 6115	68.0 ± 8.72	123.8 ± 10.46	12797 ± 1330
**D20C10**	236.2 ± 21.18	47962 ± 8815	61.0 ± 9.59*	130.8 ± 13.86 †	13722 ± 1826
**D20C20**	238.2 ± 25.85	48645 ± 8091	62.8 ± 8.26*	129.7 ± 13.37 ††	12467 ± 1778
**P-value**	0.67	0.33	0.02	0.03	< 0.001
Data were presented as Mean ± SD. The Kolmogorov-Smirnov test was used to determine the normality of the data distribution. To determine significance, one-way analysis of variance test and LSD post hoc test were used at a significance level of p ≤ 0.05. Significantly different from Cnt, *P < 0.05, **P < 0.01, ***P < 0.001, † Significantly different from D, †† Significantly different from D, p < 0.01, not significantly different from Cnt and D, p > 0.05. Cnt: Control group, Shm: Sham group, D: Diazinon (Diazinon group), C: Chrysin (C10, Chrysin 10 mg/kg), C20 (Chrysin 20 mg/kg), D20C10 (Diazinon 20 mg/kg and Chrysin 10 mg/kg), D20C20 (Diazinon 20 mg/kg and Chrysin 20 mg/kg)					

## 4. Discussion

In the present study, the beneficial effect of C, which is currently under investigation due to its important biological activities, was investigated on the reproductive system. Mice were initially examined in our other paper for sperm quality and fertility using C and D toxins (14). The results of spermatogenesis in the group that received D were significantly decreased compared to the control group. In the 2 groups where C 10 and 20 were used together with D, although it could not significantly reduce the amount of damage compared to the control group, it increased significantly compared to the D group.

In this study, the height of the embryonic epithelium of the spermatogenic tubules in the groups receiving D 20, D 20 + C 10 and D 20 + C was significantly reduced compared to the control group. The results showed that D alone can significantly reduce the height of the embryonic epithelium. Also, the lumen diameter of the seminiferous tubules in the group receiving D alone was significantly different from the control group. The lumen diameter in the groups receiving D together with C 10 and 20 also showed a significant decrease compared to the D group. The cross-sectional area of the lumen of the seminiferous tubules in the group receiving D 20 alone was significantly different from the control and Sham groups, but no significant difference was observed with the other experimental groups. These results are consistent with the study by Campos et al. who investigated the effects of C (15).

The role of genes in the SPI process is very important. The *Dazl* gene is particularly affected by the proliferation and differentiation of germ cells. In one study, the expression of this gene in the group that received only D was significantly lower than in the control group, indicating impaired germ cell division and reduced sperm count. Also, impaired expression of this gene can negatively affect cell differentiation. The results indicate that toxins such as D can disrupt the SPI process. The combination of C with D could not completely reduce the damage, but had a positive effect compared to the group that received only D (16).

The *Akap4* gene is also one of the most abundant proteins in the fibrous sheath of sperm. Of course, the physiological role of the fibrous sheath and its related proteins has not yet been clearly understood. However, some researchers believe that men lacking the *Akap4* gene are usually infertile, and their sperm count and sperm survival are greatly reduced. It is also possible that spermatozoa with a short tail may be produced in the absence of expression of this gene, leading to infertility (17). Therefore, with low expression or lack of expression of this gene, one should expect the production of spermatozoa that do not have a normal structure (18). Of course, our study did not necessarily agree with the observations of others. The expression of the *Akap4* gene in the group that received D20 alone was higher than in the control group, and this difference was statistically significant (p < 0.001). Also, the expression of this gene in the D20C10 and D20C20 groups did not change much compared to the control group. The expression of *Akap4* gene in the D20C10 group was also lower than that in the D20 group, which was also statistically significant (p < 0.05). Therefore, its effect on the SPI process cannot be challenged or ineffective.

The *Tsga10* gene also affects SPI and is expressed in the late stages of SPI. This gene plays an important role in the fibrous coating of the sperm tail and its lack of correct expression leads to azoospermia. Some researchers have concluded that overexpression of the *Tsga10* gene under normal conditions causes autophagy (19). Other researchers have suggested that decreased *Tsga10* expression reduces autophagy and increases ROS levels, which could lead to impaired spermatid differentiation, maturation, and the formation of sperm with abnormal morphology (9). In our study, the *Tsga10 *gene showed lower expression in the group that received D alone compared to the control group, which was statistically significant. Therefore, it can be concluded that D, as a damaging factor, affects the process of SPI. Moreover, the expression of this gene in the groups that received D20C10 and D20C20 showed significantly lower expression than both the control group and the group that received D alone. The results obtained can indicate that C could not inhibit the adverse effect of D on reducing the expression of the *Tsga10 *gene or its level. Therefore, its result can be associated with the ineffectiveness of C on the expression of this gene.

Research concluded that the protamine gene is related to fertility. A group of researchers concluded that if certain factors affect the expression of the protamine gene and sperm parameters in azoospermia mouse model, a decrease in the level of protamine expression compared to the normal state can lead to azoospermia. It was also found that protective elements can partially prevent damage to testicular tissue and inhibit further reduction in *protamine* gene expression (20). In line with the information obtained from various articles, we also concluded in our study that D can, as a damaging agent, cause the expression of the *protamine* gene to be disrupted and significantly reduced. Therefore, it is not unlikely that various toxins used in environmental conditions to control various pests leave various side effects, including reduced fertility in men who are exposed to them. Although one of the goals of this study is to achieve elements that can reduce the severity of the damage of such elements or to inhibit it. Therefore, the use of C is a kind of testing of protective elements that may have such an effect. However, in practice, it is observed that the prevention of damage is not very significant, and due to its lack of significance a reliable result cannot be obtained.

### Strengths and limitations

The use of medicinal plants as a diet is one of the ways to improve male fertility. Therefore, C is a flavonoid that can be extracted from medicinal plants and is available, which has been able to reduce SPI, TDI, RI, epithelial height, ductal diameter, and surface, and some genes involved in spermatogenesis, which can be our strength in this study. One of our weak points in this study was not using the expression of more genes of the spermatogenesis pathway and also not examining the results of interventions in the next generation of rats, which could not be further investigated due to higher costs. It is hoped that the current results will pave the way for further investigations from the point of view of the protein mechanism and genes of the spermatogenesis pathway with herbal therapeutic approaches.

## 5. Conclusion

According to the results, C is a flavonoid that can be extracted from medicinal plants and is available, which has been able to greatly reduce the adverse effects caused by D and possibly similar toxins. The results showed that the mean SPI, TDI, RI, epithelial height, diameter, and luminal area, and some genes involved in spermatogenesis in the control group showed a significant difference. According to the results of this research and similar research, it is suggested to carry out additional studies to know as much as possible about the medicinal properties of plants with antioxidant properties, such as C, in the treatment of male infertility problems, from the oral administration of these valuable medicinal plants in diets that it is given to improve fertility in men.

##  Data Availability

The datasets used and/or analyzed during the current study are available from the corresponding author upon reasonable request.

##  Author Contributions

E. Fattahi and SGhA. Jorsaraei designed the study and conducted the research. F. Mojibi, E. Fattahi, SGhA. Jorsaraei monitored, evaluated, and analyzed the research results. In addition, F. Mojibi, E. Fattahi, SGhA. Jorsaraei, S. Kazemi, and M. Gholamitabar Tabari reviewed the article. All authors approved the final article and took responsibility for the accuracy of the data.

##  Conflict of Interest 

The authors declare that there is no conflict of interest.

## References

[bib1] Ghajari Gh, Moosavi R (2022). Evaluation of the effects of diazinon toxin on some reproductive parameters in male rats. Pers M J.

[bib2] Delorenzi Schons D, Leite GAA (2023). Malathion or diazinon exposure and male reproductive toxicity: A systematic review of studies performed with rodents. Crit Rev Toxicol.

[bib3] Liu Y, Song X, Li C, Hu H, Li W, Wang L, et al (2022). Chrysin ameliorates influenza virus infection in the upper airways by repressing virus-induced cell cycle arrest and mitochondria-dependent apoptosis. Front Immunol.

[bib4] Xu X, Miao J, Shao Q, Gao Y, Hong L (2020). Apigenin suppresses influenza A virus‐induced RIG‐I activation and viral replication. J Med Virol.

[bib5] Mohammed HA, Sulaiman GM, Albukhaty S, Al‐Saffar AZ, Elshibani FA, Ragab EA (2023). Chrysin, the flavonoid molecule of antioxidant interest. ChemistrySelect.

[bib6] Abajlou SC, Tofighi A, Tolouei Azar J, Khaki AA, Razi M (2025). Combined effects of chrysin supplementation and exercise training on diabetes-induced oxidative stress and apoptosis in rat testicular tissue. Int J Fertil Steril.

[bib7] Pordel M, Baharara J, Amini E (2017). [Cytotoxic and antioxidant effect of chrysin on neonate mouse spermatogenic stem cells]. Feyz Med Sci J.

[bib8] Zhang Y, Liu Z, Yun X, Batu B, Yang Z, Zhang X, et al (2023). Transcriptome profiling of developing testes and first wave of spermatogenesis in the rat. Genes (Basel).

[bib9] Asgari R, Bakhtiari M, Rezazadeh D, Yarani R, Esmaeili F, Mansouri K (2021). TSGA10 as a potential key factor in the process of spermatid differentiation/maturation: Deciphering its association with autophagy pathway. Reprod Sci.

[bib10] Arévalo L, Merges GE, Schneider S, Oben FE, Neumann IS, Schorle H (2022). Loss of the cleaved-protamine 2 domain leads to incomplete histone-to-protamine exchange and infertility in mice. PloS Genet.

[bib11] Kumar N, Singh AK (2022). Impact of environmental factors on human semen quality and male fertility: A narrative review. Environ Sci Eur.

[bib12] Naz S, Imran M, Rauf A, Orhan IE, Shariati MA, Ul-Haq I, et al (2019). Chrysin: Pharmacological and therapeutic properties. Life Sci.

[bib13] Dibal NI, Garba SH, Jacks TW (2020). Morphological assessment of epididymal sperm in Wistar rats using different histological stains. Acta Vet Eurasia.

[bib14] Mojibi F, Jorsaraei GA, Fattahi E, Kazemi S, Gholami Tabar Tabari M (2023). [Evaluation of the protective effect of chrysin on sperm parameters on the process of spermatogenesis in rats receiving diazinon toxin]. J Anim Biol.

[bib15] Campos MS, Ribeiro NCS, De Lima RF, Santos MB, Vilamaior PSL, Regasini LO, et al (2018). Anabolic effects of chrysin on the ventral male prostate and female prostate of adult gerbils (Meriones unguiculatus). Reprod Fertil Dev.

[bib16] Li H, Liang Z, Yang J, Wang D, Wang H, Zhu M, et al (2019). DAZL is a master translational regulator of murine spermatogenesis. Natl Sci Rev.

[bib17] Fang X, Huang L-L, Xu J, Ma C-Q, Chen Z-H, Zhang Z, et al (2019). Proteomics and single-cell RNA analysis of Akap4-knockout mice model confirm indispensable role of Akap4 in spermatogenesis. Dev Biol.

[bib18] Dissanayake WMN, Heo JM, Yi Y-J (2023). Adverse effects of pesticide/metabolites on boar spermatozoa. Korean J Agr Sci.

[bib19] Rezazadeh  D, Ranjbarnejad  F, Mansouri  K, Mostafaie A, Barzegari E, Modarressi MH

[bib20] Alipourfard I, Khorshidian A, Babakhanzadeh E, Nazari M (2023). Susceptibility to azoospermia by haplotype analysis of protamine 1 and protamine 2 variants. Human Gene.

